# The Tristetraprolin Family of RNA-Binding Proteins in Cancer: Progress and Future Prospects

**DOI:** 10.3390/cancers12061539

**Published:** 2020-06-11

**Authors:** Yogesh Saini, Jian Chen, Sonika Patial

**Affiliations:** Department of Comparative Biomedical Sciences, School of Veterinary Medicine, Louisiana State University, Baton Rouge, LA 70803, USA; ysaini@lsu.edu (Y.S.); jchen10@lsu.edu (J.C.)

**Keywords:** post-transcriptional regulation, RNA-binding proteins, tumor suppressor, tristetraprolin, ZFP36

## Abstract

Post-transcriptional regulation of gene expression plays a key role in cellular proliferation, differentiation, migration, and apoptosis. Increasing evidence suggests dysregulated post-transcriptional gene expression as an important mechanism in the pathogenesis of cancer. The tristetraprolin family of RNA-binding proteins (RBPs), which include Zinc Finger Protein 36 (ZFP36; commonly referred to as tristetraprolin (TTP)), Zinc Finger Protein 36 like 1 (ZFP36L1), and Zinc Finger Protein 36 like 2 (ZFP36L2), play key roles in the post-transcriptional regulation of gene expression. Mechanistically, these proteins function by binding to the AU-rich elements within the 3′-untranslated regions of their target mRNAs and, in turn, increasing mRNA turnover. The TTP family RBPs are emerging as key regulators of multiple biological processes relevant to cancer and are aberrantly expressed in numerous human cancers. The TTP family RBPs have tumor-suppressive properties and are also associated with cancer prognosis, metastasis, and resistance to chemotherapy. Herein, we summarize the various hallmark molecular traits of cancers that are reported to be regulated by the TTP family RBPs. We emphasize the role of the TTP family RBPs in the regulation of trait-associated mRNA targets in relevant cancer types/cell lines. Finally, we highlight the potential of the TTP family RBPs as prognostic indicators and discuss the possibility of targeting these TTP family RBPs for therapeutic benefits.

## 1. Introduction

In healthy cells, expression of mRNAs for genes associated with cell survival pathways is maintained at normal levels through tight transcriptional and post-transcriptional mechanisms. In contrast, tumor cells possess abnormally stable mRNAs for various categories of pro-survival genes, including protooncogenes, tumor suppressors, and cytokines. A large number of these tumor-associated pro-survival mRNAs possess AU-rich elements (AREs) in their 3′-untranslated regions (3′UTRs). Specific ARE-binding proteins, such as the tristetraprolin family of RNA-binding proteins, are known to post-transcriptionally regulate the expression of these mRNAs.

The tristetraprolin family of RNA-binding proteins (TTP family RBPs) are characterized by the presence of one or more CCCH zinc finger domain(s) that contain three cysteine (C) and one histidine (H) residues. There are three human members in this family, including Zinc Finger Protein 36 (ZFP36) or TTP itself, encoded by the *ZFP36* gene; Zinc Finger Protein 36 Like 1 (ZFP36L1), encoded by the *ZFP36L1* gene; and Zinc Finger Protein 36 Like 2 (ZFP36L2), encoded by the *ZFP36L2* gene [1] (Table 1). A fourth member, Zinc Finger Protein 36 Like 3 (ZFP36L3), is restricted to rodents. Orthologues of the TTP family RBPs have been found in many vertebrates with the only exception in birds [2].

Through a highly conserved zinc finger domain, the TTP family RBPs bind to AREs at the 3′UTRs of their target mRNAs in a sequence- and structure-specific manner and catalyze the removal of the poly (A) tail, thus resulting in their mRNA decay. The consensus sequence of AREs in the 3′UTRs of the target mRNAs is UUAUUUAUU, although some variations of this sequence still mediate high affinity binding [3]. All the mammalian TTP family members appear to act similarly in biochemical studies involving RNA binding and decay. Interestingly, germline gene knockouts of the three TTP family RBPs in mice resulted in vastly different phenotypes [4,5,6,7]. For instance, while germline deletion of TTP resulted in a systemic inflammatory syndrome [4], germline deletion of ZFP36L1 was embryonically lethal [5], and germline deletion of ZFP36L2 resulted in post-natal mortality within two weeks post-birth due to defects in hematopoiesis [6]. These experiments clearly demonstrated that the TTP family RBPs may have differential target and cell/tissue-type specificity under varying physiological and pathological conditions. Furthermore, the TTP family RBPs may be expressed at different times during pre- and post-natal life. Some studies, including our unpublished observations, have also reported on the redundant functions of the TTP family RBPs [8].

Although the TTP family proteins were discovered more than 20 years ago, most of the studies investigating their role in carcinogenesis have been reported within the last decade. The TTP family RBPs have tumor-suppressor properties, which are directly related to their ability to post-transcriptionally regulate oncogenic mRNAs. For instance, oncogenes, including *NOTCH1*, *MYC*, *BCL-2*, and *COX-2*, contain 3′UTR AREs, and have been identified as direct TTP family RBP targets [8,9,10,11]. Conversely, TTP expression is also directly suppressed by certain oncoproteins [9]. TTP has also been shown to complement the function of tumor suppressors, such as p53, through downregulation of the oncogenes [12]. In fact, TTP expression is induced by p53 in cancer cells [12]. In rare instances, the TTP family RBPs are also known to directly target tumor suppressors. For instance, TTP has been shown to target the mRNA encoding the tumor suppressor LATS2 [13]. In sum, while increasing evidence suggests a protective role of the TTP proteins in tumorigenesis, some mechanisms seem to exist that counter the beneficial aspects of the TTP family RBPs.

Alterations in the expression/activity of the TTP family RBPs have been reported to be associated with multiple cancers [14] (Table 2). Numerous studies have specifically reported a loss of TTP family RBP expression in a variety of cancers [15,16,17,18]. Loss of expression/activity of the TTP family RBPs is expected to result in increased stability of their target mRNAs. Three different mechanisms for loss of expression/activity of the TTP family RBPs have been reported: (1) MicroRNA-mediated regulation; (2) epigenetic silencing via DNA methylation; and (3) modulation of protein activity through post-translational modifications, particularly phosphorylation. Regardless of the mechanisms involved, owing to the regulation of a broad range of target mRNAs concurrently, the TTP family RBPs loss can result in significant changes in gene expression and can have dramatic consequences for the development and progression of cancer.

In this review, we will discuss the key molecular traits of cancer that the TTP family RBPs regulate; the molecular mechanisms of the regulation; and the TTP family RBP mRNA targets that have been identified in various cancer cells and tissues. Specifically, the molecular traits of cancers, including uncontrolled cellular proliferation in the absence of external growth signals, resistance to apoptosis, sustained angiogenesis, as well as tissue invasion and metastasis, will be discussed. We will also discuss the potential of the TTP family RBPs as prognostic biomarkers and the possibility of targeting the TTP family RBPs for therapeutic purposes. The outstanding questions that remain will also be highlighted. 

## 2. TTP Family Proteins and Cell Cycle Control

Dysregulation of the cell cycle is a characteristic feature of all cancers. The cell cycle involves four distinct phases, i.e., G1 (Gap 1 or first growth phase), S (DNA replication phase), G2 (Gap 2 or second growth phase), and M (mitosis phase). Regulatory mechanisms are in place to ensure that cells in the G1 phase that acquire DNA damage are prohibited from entering the S phase, and that errors during DNA replication in the S phase are repaired in the G2 phase before the cells enter the M phase. Several oncogenic processes function by dysregulating the normal controls and checkpoints and enforcing the cells into cell cycle progression in a mitogen-independent manner. 

TTP’s role in regulating the cell cycle is linked to its ability to bind and destabilize critical cell cycle regulators. For instance, critical cell cycle regulators, namely c-Myc and cyclin D1, possess 3′UTR AU-rich elements and have been shown to be regulated by TTP [73]. C-Myc is a member of the Myc oncogene family of transcription factors that regulate cellular proliferation, differentiation, metabolism, and apoptosis, and are frequently dysregulated in human cancers [74]. Cyclin D1 is a proto-oncogene that regulates G1-S phase progression and is frequently overexpressed in cancer. Another example of a cell cycle checkpoint protein regulated by TTP is E2F transcription factor 1 (E2F1). E2F1 regulates G1-S phase progression and is frequently overexpressed in many types of human cancers. Aberrant expression of E2F1 is associated with high-grade tumors, metastases, and unfavorable patient prognosis. TTP was shown to post-transcriptionally regulate E2F1, suggesting that TTP controls cellular proliferation through the regulation of E2F1 mRNA stability [75]. Along similar lines, Xu et al. reported that TTP inhibits cellular proliferation in breast tumor cells in vitro and breast tumor growth in vivo by inducing cell cycle arrest at the S phase. TTP was found to inhibit c-Jun expression through blocking the nuclear factor kappa-light-chain-enhancer of activated B cells (NF-κB) p65 nuclear translocation, which resulted in increased expression of Wee1, a regulatory molecule that controls cell cycle transition from the S to the G2 phase [26]. 

Interestingly, resveratrol (3,5,4′-trihydroxystilbene), a naturally occurring polyphenol compound found in natural sources, including grape skin and red wine, was shown to activate TTP, resulting in downregulation of E2F1, inhibitor of apoptosis 2 (cIAP2), large tumor suppressor kinase 2 (LATS2), and lin-28 homolog A (Lin28)—all downstream targets of TTP—thus suppressing the proliferation and invasion/metastasis of colon cancer cells [39]. Hitti et al. performed a systematic analysis of ARE-mRNA expression across multiple cancer types, including invasive breast cancer, and showed that ARE-mRNAs were overrepresented and correlate with TTP expression. A cluster of 11 overexpressed ARE-mRNAs that are involved in the mitotic (M phase) cell cycle phase was found to negatively correlate with TTP expression. These ARE-mRNAs also physically interacted with TTP, indicating direct regulation. Furthermore, breast cancer patients with a high mean expression of this cluster showed poor survival [27]. These studies suggested an anti-mitotic role of TTP. TTP was also shown to directly bind NEDD9, a protein that has a potential role in prostate cancer cell growth regulation [63].

Chen et al. showed that cyclin-dependent kinase 6 (CDK6) is post-transcriptionally regulated by ZFP36L1. These authors demonstrated that ZFP36L1 functions as a positive regulator of monocyte/macrophage differentiation by regulating CDK6. Accordingly, levels of ZFP36L1 were found to be significantly reduced in acute myeloid leukemia patients [52]. ZFP36L1 has also been suggested to be a post-transcriptional regulator of the cell cycle signaling genes, including *E2F1* and *CCND1* (cyclin D1). In a recent study, ZFP36L1 was particularly shown to regulate hypoxia signaling through direct binding and degradation of HIF1A. This study indicated a tumor-suppressor role for ZFP36L1 through regulation of hypoxia, cell cycle, and angiogenesis [15]. Furthermore, these authors found that ZFP36L1 is epigenetically silenced through hypermethylation of the second exon and is downregulated in several patient cohorts of bladder and breast cancers. Functionally, silencing ZFP36L1 enhanced tumor cell growth while overexpression of ZFP36L1 suppressed cell proliferation and migration in bladder and breast cancer cell lines [15]. Finally, both ZFP36L1 and ZFP36L2 were shown to inhibit cellular proliferation through downregulation of cyclin D expression, resulting in cell cycle arrest at the G1 phase [76]. All these studies indicate that TTP, ZFP36L1, and ZFP36L2 are critical regulators of the cell cycle.

## 3. TTP Family Proteins and Control of Apoptosis

One of the hallmark characteristics of cancer is to evade apoptosis or resist cell death [77]. Apoptosis occurs through two distinct pathways: the intrinsic (mitochondrial) pathway and the extrinsic (death receptor) pathway. While the two pathways are distinct, both involve activating caspases in the final steps.

The TTP family RBPs modulate tumor cell apoptosis by directly regulating the apoptotic mediators within both pathways. Johnson et al. demonstrated that TTP results in apoptotic cell death in various cell types, likely through direct regulation of the TTP targets [78]. This was one of the earliest studies that indicated a role of TTP proteins in cell survival and apoptosis. The authors suggested that TTP was unique and somewhat different from the two other family members, ZFP36L1 and ZFP36L2, because TTP, but not ZFP36L1 and ZFP36L2, could also sensitize the cells to apoptosis by inducing TNFα. However, it remained unclear whether TTP was inactive upon ectopic expression in these studies. Hydroquinone, an aromatic organic compound, induces apoptosis in human leukemia U937, human leukemia HL-60, and Jurkat cells through a TTP-dependent mechanism. Mechanistically, this study showed that TTP phosphorylation and inactivation through the p38 MAPK pathway resulted in increased TNFα-induced apoptosis [50]. Along similar lines, albendazole, a microtube-targeting anthelmintic, was demonstrated to induce apoptosis in human leukemia cells through the p38–TTP–TNFα axis [51].

Conversely, resveratrol was able to induce TTP expression in human glioma cells that resulted in apoptosis and suppression of cell growth through destabilization of the urokinase plasminogen activator (uPA) and urokinase plasminogen activator receptor (uPAR). Both, uPA and uPAR are overexpressed in glioblastomas and play a role in invasion [47].

PIM1, an oncogenic serine-threonine kinase, functions by repressing apoptosis and is a direct target of TTP [61,79]. In fact, ectopic TTP expression impaired the viability and invasiveness of glioblastoma multiforme cancer cells by destabilizing the PIM1, PIM2, and X-linked inhibitor of apoptosis proteins (XIAP) in these cells [45]. Park et al. recently reported that TTP enhances cisplatin sensitivity in head and neck squamous cell carcinoma (SCCHN) cells by reducing the levels of BCL-2, an anti-apoptotic protein, which is overexpressed in cancer and confers resistance to cisplatin [70]. While, earlier, Lee et al. had showed that ZFP36L1 enhanced cisplatin sensitivity in SCCHN cells by inhibiting the human inhibitor of apoptosis protein-2 (cIAP2) and resulting in increased caspase-3 activity [71]. ZFP36L1 has also been shown to mediate its pro-apoptotic effects on malignant B-cells by regulating BCL-2 [10]. The BCL-2 family of proteins control the permeabilization of the mitochondrial outer membrane, thus regulating commitment to apoptosis. The role of ZFP36L2 in modulating apoptosis remains unexplored. Together, all these studies indicate an important role of the TTP family RBPs in regulating apoptosis.

## 4. TTP Family Proteins and Regulation of Pro-Tumorigenic Inflammatory Mediators

TTP is a known regulator of inflammation. This critical function of TTP first became evident when germline TTP knockout mice were generated [4]. TTP knockout mice appeared normal at birth; however, within 2–3 weeks, they developed a systemic inflammatory syndrome characterized by cachexia, arthritis, dermatitis, conjunctivitis, myeloid hyperplasia, and autoimmunity [4]. This phenotype was largely attributed to overexpression of TNFα, a potent pro-inflammatory cytokine, as evidenced by the prevention of the development of the syndrome by anti-TNFα antibody injections [4]. Further, it was demonstrated that TTP binding to AREs within the 3′UTR of TNFα mRNA results in an increased turnover, an effect that was abrogated in TTP deficiency [80]. Subsequent studies showed that a number of other pro-inflammatory cytokines and chemokines, including IL-23, IL-17, IL-1β, CXCL1, and CXCL2, are also directly regulated by TTP [81,82]. These and other mRNA targets of the TTP family RBPs are reviewed elsewhere [83]. Furthermore, germline overexpression of TTP using its endogenous promoter resulted in protection against immune-mediated inflammatory diseases, including arthritis, psoriasis, and autoimmune encephalomyelitis [84]. These critical studies indicate that TTP directly regulates inflammation and that loss of TTP expression/activity results in enhanced inflammation.

Inflammation is also a critical component of the tumor progression and tumor microenvironment, and many tumors are known to arise at the site of chronic inflammation. TTP is a well-established post-transcriptional regulator of pro-inflammatory cytokines and chemokines and, due to this function, TTP is an important modulator of tumor development and progression. Twizere et al. showed that TTP physically interacts with viral protein Tax, thus reverting the inhibition of pro-inflammatory cytokine TNFα [48]. The authors indicated that this may be of importance in cell transformation caused by leukemogenic viruses. Stoecklin et al. demonstrated the tumor-suppressor role of TTP in a v-H-ras-dependent mast cell tumor model through regulation of interleukin-3 (IL-3) [56]. Tumor cells in this model exhibit abnormally stable expression of IL-3 mRNA as part of an oncogenic autocrine loop. TTP delayed tumor progression by four weeks by enhancing IL-3 mRNA degradation in this model. Interestingly, TTP expression was lost in tumors that managed to appear at or after four weeks. Additionally, TTP reduced the cloning efficiency in vitro when transfected into a fully established tumor cell line and the growth of the inoculated cells in vivo. These studies indicate a critical role of mRNA stabilization in oncogenesis and suggest that tumor suppression is achievable by interfering with mRNA turnover.

Sawaoka et al. showed an interesting mechanism of regulation of cyclooxygenase-2 (COX-2) by TTP in colon adenocarcinoma cells. These cells express two distinct transcript variants of COX-2: a full length, 4465nt mRNA; and a truncated 2577nt polyadenylation variant, in which the terminal 1888 nt 3′untranslated region is absent. During cellular growth, the levels of the full-length transcript reduced, whereas the levels of the truncated variant increased. Most importantly, TTP levels were inversely correlated with the levels of the full-length transcript, and TTP transfection resulted in a reduction in the levels of the full-length transcript, indicating TTP regulation of COX-2 in these cells [11]. COX-2 is a product of an immediate early gene that is induced by growth factors and cytokines and plays a role in cellular proliferation [85,86,87]. COX-2 expression is increased in a number of cancers, including human colorectal [88], esophageal [89], pancreatic [90], lung [91], prostate [92], and mammary [93].

Similarly, TTP was shown to destabilize interleukin 8 (IL-8) and vascular endothelial growth factor (VEGF) mRNAs in malignant glioma cells, resulting in a dose-dependent decrease in cellular proliferation, loss of cell viability, and apoptosis. TTP was, in fact, ubiquitously expressed in primary gliomas and benign astrogliotic tissues; however, hyperphosphorylated/inactive TTP was present in malignant glioma tumors. It is generally accepted that the TTP activity is repressed upon phosphorylation [46]. Al-Souhibani et al. showed that TTP expression is significantly lower in invasive breast cancer cells compared to normal breast cells, and that the genes involved in cellular growth, invasion, and metastasis, namely matrix metalloproteinase 1 (MMP1), urokinase-type plasminogen activator (uPA), and urokinase plasminogen activator receptor (uPAR), were directly regulated by TTP in breast cancer cells [21]. Along the same lines, TTP was found to be weakly expressed in melanoma cells. These cells express high levels of the C-X-C motif chemokine ligand 8 (CXCL8), which plays a role in cellular growth and angiogenesis. These authors further demonstrated that extracellular signal-regulated kinase (ERK) inhibition restored TTP, which destabilized and inhibited CXCL8, suppressed cellular proliferation, and induced apoptosis [57]. TTP was also shown to directly regulate hypoxia-inducible factor 1 (HIF-1), a factor critically required for survival in hypoxic conditions, indicating that a low TTP poses a significant advantage to cancer cells by increasing HIF-1 and allowing adaptation to hypoxia [94]. Interestingly, latent membrane protein 1, a viral oncoprotein, was found to significantly enhance HIF-1A expression in nasopharyngeal carcinoma cells by inhibiting TTP [95].

In yet another study, TTP was shown to post-transcriptionally regulate interleukin 23 (IL-23) in mouse colon cancer cells [35]. IL-23 is highly expressed in many tumors and its levels correlate with tumor progression. Squamous cell carcinoma of the head and neck (SCCHN) patients with low interleukin 6 (IL-6) and high MMP9, or with high IL-6 and low MMP9, were found to have the poorest outcomes followed by patients with both high IL-6 and high MMP9. In comparison, patients with low IL-6 and low MMP9 had the best outcomes with respect to tumor recurrence, surgery, or death. Functionally, TTP suppression enhanced cellular invasiveness in vitro in an oral-cancer-equivalent 3D model and in vivo in chick chorioallantoic membrane models, resulting from increased secretion of IL-6, MMP2, and MMP9 [68].

TTP was found to be remarkably reduced in gastric cancer and inversely correlated with interleukin 33 (IL-33) expression. Furthermore, low TTP expression contributed to gastric cancer progression and was associated with depth of invasion, lymph node metastasis, advanced TNM stage, and poor survival. Conversely, elevated TTP expression was shown to inhibit the proliferation, migration, and invasion of gastric cancer cells through suppression of IL-33, a tumor promoting cytokine [43]. Similar results were found in human glioma tissues and cells where TTP was significantly downregulated and associated with reduced survival. In this particular study, TTP inversely correlated with IL-13 levels in glioma tissues and TTP inhibited the growth, migration, and invasion of glioma cells through downregulation of IL-13 and attenuation of the PI3K/Akt/mTOR pathway [96].

TTP knockout mice has increased numbers of cytotoxic T-cells due to direct regulation of interleukin 27 (IL-27), a CD8 + T-cell regulatory cytokine. Interestingly, in a mouse mammary gland tumor model, TTP knockout mice showed retracted tumor growth due to increased tumor-infiltrating CD8+ T cells [29]. Kratochvill et al. showed that TTP is constitutively highly expressed in tumor-associated macrophages. However, the effects of TTP on mRNA stability were blocked by the constitutively active p38 in the tumor microenvironment, which drove the production of inflammatory cytokines [97]. A very elegant study by Coelho et al. showed innately immunoresistant RAS mutant tumors are characterized by the upregulation of immunosuppressive protein programmed death-ligand 1 (PD-L1) through RAS–MEK–MK2-induced TTP phosphorylation/inactivation, resulting in increased PD-L1 mRNA stability. In humans, RAS activation was associated with PD-L1 upregulation in human lung and colon adenocarcinoma [37]. TTP has also been identified as one of the eight genes functionally related to the NF-κB pathway that were highly downregulated in lethal prostate cancer [63]. Gambogic acid, a polyprenylated xanthone, was demonstrated to significantly inhibit cancer stem cells in colorectal carcinoma, both in vitro and in vivo, by inhibiting EGFR–ERK signaling, resulting in upregulation of TTP [38]. TTP has also been demonstrated to be a post-transcriptional regulator of aryl hydrocarbon receptor repressor (AHRR) in breast cancer cells [23].

Similar to TTP, ZFP36L1 phosphorylation and inactivation by the p38–MK2 axis has been shown to stabilize Nanog and Klf4 in triple-negative breast cancer cells, resulting in breast cancer stem cell phenotype, a feature of chemotherapy-resistance in triple-negative breast cancer [31].

## 5. TTP Family Proteins and Cellular Senescence

Cellular senescence is characterized by cells undergoing growth arrest in response to a wide variety of extrinsic and intrinsic insults, including DNA damage, loss of telomeres, and oncogenic activation. Cells undergoing senescence secrete a collective set of proteins that includes cytokines, chemokines, and growth factors, among others. While under basal conditions, cellular senescence may be beneficial in maintaining tissue homeostasis, cellular senescence is potentially detrimental in aging. Cellular senescence has dynamic roles in cancer: beneficial in tumor cells by improving the therapeutic outcomes, and detrimental in non-tumor cells by causing relapse and secondary tumors. Selected studies have shown a role for the TTP family proteins in regulating senescence. For instance, human papilloma virus-18 (HPV-18)-positive HeLa cells were used to show that TTP promoted cellular senescence through rapid decay of E6-associated protein (E6-AP) mRNA, resulting in p53 stabilization and inhibition of human telomerase reverse transcription gene (*hTERT*) transcription. E6 is a viral protein that HPV uses for cellular transformation. Association of E6 with E6-AP facilitates cell transformation by p53 degradation and activation of *hTERT*. This study linked the TTP-mediated post-transcriptional regulation to HPV-associated cervical carcinogenesis [34]. In fact, TTP was found to be consistently absent in cervical carcinomas compared to normal human cervixes. These studies also suggested the tumor suppressive role of TTP in cervical cancer.

In a recent study, ZFP36L1 was demonstrated as a key regulator of cellular senescence by directly regulating components of the senescence-associated secretory protein (SASP) through post-transcriptional regulation. In this study, ZFP36L1 was found to signal downstream of mTOR in regulating SASP mRNAs and phosphorylation inhibited ZFP36L1 activity [98]. The role of ZFP36L2 in cellular senescence remains undetermined.

## 6. TTP Family Proteins and Regulation of Angiogenesis

One of the hallmarks of cancer is angiogenesis, the formation of new blood vessels, which are required for supplying nutrition and overcome a hypoxic microenvironment in rapidly growing tumor masses. VEGF is an angiogenic cytokine that plays a key role in tumor angiogenesis. VEGF and IL-6 are markedly increased in squamous cell carcinoma of the head and neck (SCCHN) and are associated with poor survival. GALR2, a pro-survival G-protein coupled receptor promoted angiogenesis via p38-mediated phosphorylation/inactivation of TTP, resulting in increased VEGF and IL-6 levels both in vitro in SCCHN cancer cells and in vivo in murine tumor xenografts and chorioallantoic membrane models [69].

Importantly, ZFP36L1 has been specifically shown to post-transcriptionally regulate VEGF [99]. Interestingly, a single intratumoral injection of a ZFP36L1 fusion protein was shown to be effective at decreasing VEGF, acidic FGF, TNFα, IL-1α, and IL-6, as well as at reducing tumor growth [100]. Both TTP and ZFP36L1 have been demonstrated to regulate HIF1α, a member of the family of transcription factors that are the primary effectors of the adaptive response of tumor cells to hypoxia [15,101]. The role of ZFP36L2 in regulating modulators of angiogenesis has not been explored yet.

## 7. TTP Family Proteins and Epithelial Mesenchymal Transition

Epithelial–mesenchymal transition (EMT) is a reversible process whereby epithelial cells transition to the mesenchymal phenotype by repressing epithelial-specific traits, i.e., intercellular adhesion and proliferation, and acquisition of mesenchymal traits, i.e., migration and invasion. EMT is a crucial step in metastasis and drug resistance, and epithelial tumors are well known to undergo EMT. TTP has been shown to directly regulate EMT regulators, including ZEB1 (zinc finger E-box binding homeobox 1), SOX9 (sex-determining region Y box 9), and MACC1 (metastasis associated in colon cancer 1), all of which are known to be downregulated in colorectal carcinomas (CRC). Re-expressing TTP reverted the EMT phenotype in this study [36]. Two other EMT regulators, TWIST1 (twist-related protein 1) and SNAIL1 (zinc finger protein snail 1), are also known targets of TTP [36]. Interestingly, TTP was identified as a target of a microRNA, miR-29a, and miR-29a-mediated downregulation of TTP was associated with EMT and metastasis in breast cancer [25].

Rataj et al. demonstrated that ZFP36L1 was markedly suppressed in breast cancer cells and patient tissues and that a derivative of ZFP36L1 fused to cell-penetrating peptide inhibited the proliferation, migration, invasion, and anchorage-independent growth in vitro and impaired the tumor growth and EMT markers, including Snail, Vimentin, and N-cadherin, in vivo [32]. Interestingly, ZFP36L1 was identified as a key regulator of neural progenitor cell-fate transition from oligodendrocyte to astrocytes and through this process it is a key regulator of processes such as myelination and gliomagenesis [49]. This study showed that while the loss of ZFP36L1 in the neural lineage resulted in myelination deficits due to the oligodendrocyte–astrocyte switch, in tumorigenesis this process was in fact beneficial by preventing gliomagenesis, thus enhancing survival. The role of ZFP36L2 in EMT has not been explored yet.

## 8. TTP Family Proteins and Tumor Suppressor and Oncogenic Roles

Tumorigenesis is a consequence of mutations in oncogenes and tumor-suppressor genes that frequently result in either an overexpression of oncogenes or loss of tumor suppressors. A key study in 2012 discovered TTPs important role as a tumor suppressor. The MYC oncoprotein was found to directly suppress TTP transcription, and TTP repression appeared to be a hallmark of malignancies with MYC involvement. Furthermore, enforced expression of TTP impaired the development of lymphoma and abolished the maintenance of the malignant state. ZFP36L1 was also repressed by MYC; however, it was not suggested to be a tumor suppressor in this model [9].

TTP has also been shown to function as a tumor suppressor through downregulation of estrogen receptor alpha (ER-α) transactivation, resulting in reduced cellular proliferation and reduced potential of the cells to form tumors in a mouse model. In this study, TTP was shown to be associated with ER-α and was recruited to the promoter region, indicating that TTP may be a bona fide nuclear receptor corepressor [24]. TTP has also been shown to be downregulated in hepatocellular carcinoma (HCC) cells and tumors through an epigenetic mechanism that involves hypermethylation of a single CpG site within the TGFβ1 responsive region of the TTP promoter. The epigenetic inactivation of TTP resulted in an increased half-life of c-Myc, causing cancer cells to undergo selective resistance to TGFβ1 antiproliferative signaling [53]. TTP is significantly downregulated in liver tumors. During tumor progression, TTP functions as a tumor suppressor and inhibits proliferation and migration, reduces expression of several oncogenes, and increases chemo sensitivity [54]. The anti-proliferative properties of metformin, an anti-diabetic drug, in breast cancer cells were mediated by induction of TTP through c-Myc downregulation [28].

Interestingly, ZFP36L1 was found to be downregulated due to enhancer hypermethylation within the second exon in myelofibrosis, which conversely led to an increased expression of its target mRNAs. Functionally, ZFP36L1 expression induced apoptosis in leukemia cells, indicating a tumor-suppressor role of ZFP36L1 in myelofibrosis [59]. Similar to TTP and ZFP36L1, ZFP36L2 also functions as a tumor suppressor. Hypermethylation of a super-enhancer site in ZFP36L2 resulted in epigenetic silencing in a large data set of esophageal squamous cell carcinoma (SCC) whole-exome sequenced tissues. This phenomenon was also found in other SCCs analyzed from the cancer genome atlas (TCGA) and resulted in reduced mRNA expression in all SCCs [41].

The strongest evidence for the role of ZFP36L1 and ZFP36L2 as tumor suppressors came from studies done by Hodson et al. [8]. These authors demonstrated that loss of both ZFP36L1 and ZFP36L2 in mouse thymocytes resulted in the development of T cell acute lymphoblastic leukemia (T-ALL) due to stabilization of an oncogenic transcriptional regulator, Notch 1 [8]. Interestingly, both ZFP36L1 and ZFP36L2 were found to function in a redundant manner in this study. Recently, genomic mutation in *ZFP36L1* was identified as a potential driver of tumorigenesis in patients with concomitant diffuse large B-cell lymphoma and hepatitis B virus (HBV) infection [55]. One study in particular suggested an oncogenic role for ZFP36L2. The authors showed that tandem duplication induced amplification of the super enhancers and were associated with an increase in ZFP36L2 expression in ~10% of gastric cancers. Functionally, ZFP36L2 promoted the growth of gastric cancer cells in this study [44]. Together, these studies indicate that all three members of the TTP family RBPs function as tumor suppressors in various types of cancers.

## 9. TTP Family Proteins and Regulation of Tumor Metastasis

Tumor metastasis is defined by cancer cells acquiring features of motility, invasion, plasticity, and ability to colonize secondary organs/tissues, and is the primary cause of cancer morbidity and mortality. Interestingly, microRNA-29a (miR-29a) was found to promote tumor progression and invasion by downregulating TTP both in vitro and in vivo in pancreatic cancer. miR-29a was upregulated and TTP was downregulated in pancreatic cancer cells and tissues [60]. In another study, two main clusters of breast cancers that differed on their lymph node status were identified from the breast cancer serial analysis of gene expression. Interestingly, ZFP36L1 was upregulated only in lymph node positive primary breast cancer, indicating that patterns of gene expression in primary tumors at the time of surgical removal could discriminate those that have lymph node metastasis [30]. Finally, ZFP36L2 was identified as an NME1, a metastatic suppressor, regulated gene in a screen of two metastatic cancer cells: melanoma and follicular thyroid carcinoma [42].

## 10. TTP Family Proteins as Potential Biomarkers

A very elegant study in 2010 showed that TTP is widely suppressed in a number of human cancers, including those of the thyroid, lung, ovary, uterus, and breast, as well as in a number of cancer cell lines, including those of lung and cervical cancer. Here, suppressed TTP was a negative prognostic indicator in breast cancer where more advanced tumors exhibited the weakest TTP expression [16]. Moreover, restoring TTP expression in cancer cells resulted in suppression of the tumorigenic phenotypes while reducing the TTP levels promoted the neoplastic phenotype [16]. Another similar study showed that among breast cancer types, higher grade tumors showed the weakest TTP expression at the protein level compared to low grade tumors, suggesting that the TTP protein levels correlate with prognosis [17].

TTP has also been suggested as a promising biomarker for prostate cancer risk assessment. TTP expression was markedly reduced in metastatic prostate cancer compared to primary tumors [64]. Men with low TTP-expressing primary prostate cancer had significantly increased chances of biochemical reoccurrence in this study. Induction of TTP inhibited the growth, proliferation, and tumorigenic potential of prostate cancer cells in a mouse xenograft model of prostate cancer [64]. Another study that also investigated prostate cancers, showed that low-TTP tumors had faster reoccurrence or metastasis versus high-TTP tumors [66]. Additionally, the low time to reoccurrence in low-TTP tumors was more pronounced in low-grade tumors. This study suggested that TTP is a promising prostate cancer biomarker for predicting the low-grade radical prostatectomy prostate cancer patients that will have poor outcomes [66]. Another study similarly showed low TTP expression in prostate cancer compared to non-cancerous tissues [65].

Low TTP expression in tumors versus adjacent normal tissues was also shown in pancreatic cancer [61]. Here, TTP expression was almost negative in poorly differentiated cancer, weakly positive in moderately differentiated, and highly positive in well differentiated pancreatic cancers. Low TTP expression was associated with age, tumor size, tumor differentiation, postoperative T stage, postoperative N stage, and TNM stage. Low TTP expression correlated with low patient survival rates and poor prognosis, suggesting that TTP could act as a prognostic indicator in pancreatic cancer [61]. Components of the AP-1 transcription factor, including JUN, JUNB, FOS, FOSB, were enriched in association with TTP as a conserved co-regulated group of genes and were significantly downregulated in breast, liver, lung, kidney, and thyroid carcinomas. Patients with low expression of these genes displayed poor prognosis [18].

Furthermore, TCGA datasets for breast cancer, lung adenocarcinoma, lung squamous cell carcinoma, and colon adenocarcinoma revealed a shared signature of 50 genes that were differentially expressed between the low- and high-TTP-expressing tumors [19]. The TTP-low gene signature was also a feature of several other cancers, including pancreatic, bladder, and prostate from non-TCGA datasets. Low TTP expression was a poor prognostic indicator in breast cancer and lung adenocarcinoma patients and was associated with decreased survival and more aggressive necrotic tumors. A TTP-low signature was characterized by perturbation of several inflammatory pathways in this study [19].

ZFP36L1 was found to be one of the genes with variants that was associated with an increased risk of subtype-specific epithelial ovarian cancers [40]. ZFP36L2 was overexpressed in pancreatic ductal adenocarcinoma (PDAC) tissues and cells as a result of suppression of microRNA-375, indicating the involvement of ZFP36L2-regulated pathways in PDAC pathogenesis. This was further supported by silencing ZFP36L2 in vitro, which inhibited cancer cell aggressiveness in PDAC cells. High ZFP36L2 expression also predicted shorter survival in PDAC, indicating that ZFP36L2 expression could be used as a prognostic marker in PDAC [62]. ZFP36L2 was also identified as a potential candidate for prediction of bone metastasis of breast cancer [33]. Finally, ZFP36L2 has been shown as a reoccurrence-associated gene in bladder cancer [20].

## 11. TTP Family Proteins and Response to Treatment

TTP proteins have also been associated with response to treatment in cancer in a few selective studies. Griseri et al. showed the presence of a synonymous polymorphism (rs3746083) in the TTP gene in an aggressive TTP-negative breast cancer cell line [22]. Interestingly, this mutation did not change the corresponding amino acid but affected the protein translation and was significantly associated with a lack of response to Herceptin treatment in HER2-positive breast cancer patients [22]. Whole genome microarray profiling of peripheral blood mononuclear cells of locally advanced rectal cancer patients revealed that, among other genes, TTP was differentially expressed between responders and non-responders to chemoradiotherapy [67]. Finally, the antitumor activity of curcumin analogue DM-1 in melanoma was shown to be mediated by multiple targets, which included TTP, ZFP36L1, and ZFP36L2, among others [58].

## 12. Outstanding Questions

A few outstanding questions remain regarding the role of the TTP family RBPs in carcinogenesis. For instance, much of our current knowledge regarding the role of TTP family RBPs in human cancer comes from the gene and protein expression data on patient tumor samples. Therefore, it is not clear whether the loss of TTP family RBPs is an early event that initiates tumor development or is a consequence of tumor development. Hence, there is an utmost need to develop transgenic animal models to understand the mechanisms by which the TTP family RBPs modulate the initiation and progression of cancer.

## 13. Conclusions

**Figure 1:**
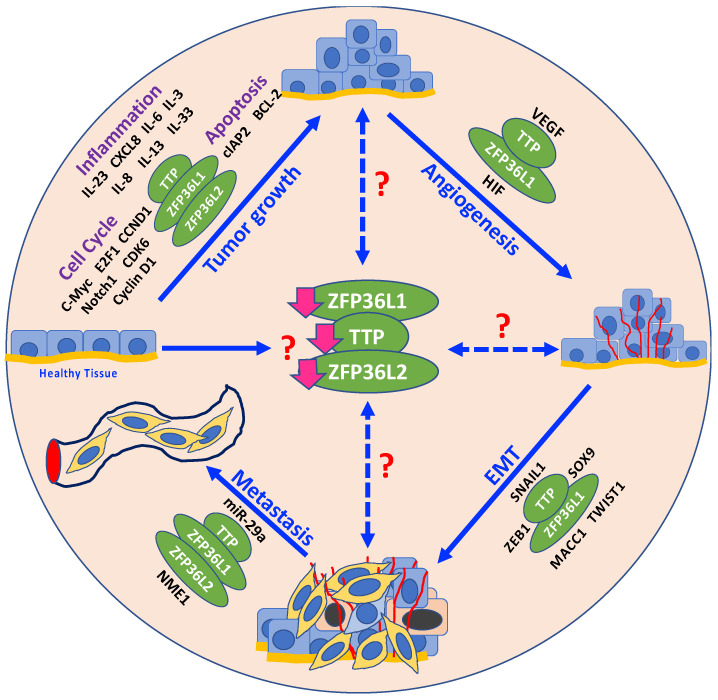
Schematic representation of the various molecular cancer traits that are potentially regulated by TTP family RBPs: TTP family RBPs regulate mRNA targets (shown in black around TTP family RBPs, represented in green ovals) at various stages during carcinogenesis, including tumor growth, angiogenesis, epithelial-mesenchymal transition (EMT), and metastasis. At what stage during the process of carcinogenesis does the loss of expression (downward pink arrows) of TTP family RBPs occur remains unknown (red question mark alongside downregulated expression of TTP family RBPs in the center of the figure). For example, it is possible that normal cells undergo loss of TTP family RBPs expression under the influence of an unknown stressor, thus driving tumor initiation or complementing the effects of the driver genes. Conversely, it is possible that loss of TTP family RBP expression occurs at subsequent stages after initial tumor formation, thus accelerating tumor progression and promoting tumor aggressiveness. Broken bidirectional arrows with red question marks represent this possibility.

Together, the studies discussed in this review indicate that the TTP family RBPs are critical regulators of multiple cancer traits (Figure 1). Given the increasing number of cancers in which TTP family proteins have been reported to be dysregulated, it appears that this family of proteins are common and important regulators of many, if not all, cancer types. It also appears that the TTP family RBPs are frequently silenced with loss of function in a majority of cancers, indicating their role as tumor suppressors. Silencing of TTP family RBP expression occurs at the epigenetic, post-transcriptional, and post-translational levels. Moreover, TTP family proteins appear to regulate multiple pathways involved in cancer development and progression and present poor prognostic outcomes for patients. While conventional cancer therapies target single genes or pathways at one time, therapeutically targeting TTP family RBPs, the master regulators of multiple cancer-relevant genes, would target multiple cancer-relevant pathways simultaneously. Therefore, targeting these proteins and restoring their function may represent an effective and novel therapeutic approach.

## Figures and Tables

**Table 1:** Human tristetraprolin (TTP) family RNA-binding proteins.

Gene Name	Protein Names	Ref-Seq mRNA	Ref-Seq Protein	Amino Acids
*ZFP36*	TTP, TIS11, GOS24, NUP475	NM_003407	NP_003398	332
*ZFP36L1*	TIS11b, cMG1, BRF1, ERF1, Berg36,	NM_004926	NP_004917	407
*ZFP36L2*	TIS11d, BRF2, ERF2	NM_006887	NP_008818	497

**Table 2:** TTP family of RNA-binding proteins in various cancers.

Cancer Type/Cancer Cell Lines	TTP Family RBPs	Major Findings (References)	Targets
Bladder Cancer	TTP/ZFP36	Bladder cancers express a TTP-low gene signature [19]	
	ZFP36L1	ZFP36L1 targets cell-cycle and hypoxia regulators, CCND1, E2F1, and HIF1A in bladder cancer cells [15].	CCND1, E2F1, HIF1A
	ZFP36L2	ZFP36L2, among other 7 genes, was identified as a prognostic indicator in muscle-invasive bladder cancer [20].	
Breast Cancer	TTP/ZFP36	TTP is significantly downregulated in invasive breast cancer cells; TTP directly targets uPA, uPAR, and MMP1 [21].	uPA, uPAR, MMP1
		TTP is downregulated in advanced breast and prostate cancers and is a negative prognostic indicator in breast cancer patients. Restoring TTP expression suppresses cell proliferation, resistance to apoptosis, and VEGF mRNA [16].	VEGF
		TTP expression inversely correlates with breast cancer aggressiveness and metastatic potential. A synonymous polymorphism in TTP gene in Hs578T cells is significantly associated with lack of response to Herceptin in HER2-positive breast cancer patients [22].	
		TTP inhibits AHRR expression [23].	AHRR
		TTP is significantly downregulated in invasive breast carcinomas. TTP expression positively correlates with differentiation in normal and tumor cells [17].	
		Low TTP-expressing breast cancer and lung adenocarcinoma patients show reduced survival and more aggressive tumors. The TTP-low gene signature is characterized by 20 underexpressed CREB targets [19].	
		TTP binds to ERα and represses ERα transactivation in breast cancer cells, resulting in reduced proliferation and reduced ability of cells to form tumors in a mouse model [24].	
		MicroRNA-29a overexpression suppresses TTP and promotes EMT and metastasis in breast cancer cells. miR-29a is upregulated and TTP is downregulated in breast cancer patient samples [25].	
		TTP inhibits c-Jun transcription by impairing NF-κB p65 nuclear translocation resulting in S-phase cell cycle arrest in breast cancer cells [26].	
		TTP suppresses mitosis by downregulating a cluster of mitosis ARE mRNAs. Poor breast cancer patient survival is significantly associated with low TTP and high mitotic ARE-mRNAs [27].	
		Metformin induces TTP expression in breast cancer cells in a Myc-dependent manner and impairs cell proliferation [28].	
		Macrophage TTP suppresses T-cell function by inhibiting IL-27 production, thus accelerating tumor growth [29].	IL-27
	ZFP36L1	ZFP36L1, among others, was overexpressed in lymph node positive primary breast tumors [30].	
		Chemotherapy induced activation of p38 resulted in phosphorylation/inactivation of ZFP36L1 thus stabilizing Nanog and Klf4 mRNA that resulted in chemotherapy-resistant breast cancer stem cell phenotype [31].	KLF4, NANOG
		ZFP36L1 is downregulated in human breast tumor samples and three breast cancer cell lines [32].	
	ZFP36L2	Expression of ZFP36L2, among other genes, significantly associated with the development of bone metastasis in breast cancer [33].	
Cervical Cancer	TTP/ZFP36	TTP is downregulated in cervical cancer tissues. In HPV-positive HeLa cells, TTP induced cellular senescence by promoting decay of the cellular ubiquitin ligase E6-associated protein (E6-AP) [34].	E6-AP
Colon, Colorectal Cancer	TTP/ZFP36	TTP binds and degrades COX-2 mRNA in human colorectal adenocarcinoma cells [11].	COX-2
		TTP binds and degrades IL-23 mRNA in mouse colon cancer cells [35].	IL-23
		Forced expression of TTP blocks EMT and induces anoikis. TTP expression impairs three key EMT transcription factors, ZEB1, MACC1 and SOX9 [36].	ZEB1, MACC1, SOX9
		TTP negatively regulates PD-L1 expression, an immunosuppressive protein that plays a role in evasion of the host immune system [37].	PD-L1
		Gambogic acid (GA) killed stem-like colorectal cancer cells by upregulating TTP [38].	
		Resveratrol suppressed the proliferation and invasion/metastasis of colorectal cancer cells by activating TTP [39].	
Epithelial Ovarian Cancer	ZFP36L1	ZFP36L1 was identified as mucinous-type epithelial ovarian cancer risk gene in a GWAS study [40].	
Esophageal Squamous cell Carcinoma	ZFP36L2	ZFP36L2 was identified as a significantly mutated gene in esophageal squamous cell carcinoma and was validated as a tumor suppressor in this cancer type [41].	
Follicular Thyroid Carcinoma	ZFP36L2	ZFP36L2 was identified as a metastasis suppressor, NME1 regulated gene in human follicular thyroid carcinoma cell lines [42].	
Gastric Cancer	TTP/ZFP36	TTP is significantly reduced in gastric cancer tissues and is associated with invasion, lymph node metastasis, and survival. TTP suppresses IL-33 and inhibits the progression of gastric cancer [43].	IL-33
	ZFP36L2	ZFP36L2 is upregulated in gastric cancer samples. Overexpressed ZFP36L2 in gastric epithelial cells promoted cell growth and colony formation. Silencing *ZFP36L2* reduces NCI-N87 growth in vivo. A tandem duplication hotspot in the super-enhancer region of *ZFP36L2* was associated with an increase in ZFP36L2 expression [44].	
Glioblastoma Multiforme	TTP/ZFP36	Ectopic TTP expression impairs the viability and invasiveness of glioblastoma cell lines. PIM-1, PIM-2, and XIAP are TTP targets [45].	PIM-1, PIM-2, XIAP
Glioma	TTP/ZFP36	Hyperphosphorylation/inactivation of TTP by p38-MAPK promoted progression of malignant gliomas by inhibiting its RNA destabilizing function. Induced expression of TTP blocked glioma cell proliferation and survival through rapid decay of IL-8 and VEGF [46].	IL-8, VEGF
		Resveratrol suppressed cell growth and induced apoptosis in human glioma cells by inducing TTP [47].	
		Retroviral oncoprotein Tax interacts with TTP and increases TNFα expression [48].	
	ZFP36L1	ZFP36L1 is required for oligodendrocyte-astrocyte lineage transition and thus is an important regulator of gliomagenesis [49].	
Leukemia	TTP/ZFP36	Hydroquinone induces apoptosis in human leukemia cells through p38 MAPK-TTP phosphorylation/inactivation and resulting TNFα upregulation [50].	TNFα
		Albendazole induces apoptosis in human leukemia cells through p38 MAPK-TTP phosphorylation/inactivation and resulting TNFα upregulation [51].	TNFα
	ZFP36L1	ZFP36L1 is downregulated in acute myeloid leukemia patient samples [52].	NOTCH1
		Thymocyte-specific ZFP36L1 and ZFP36L2 deficient mice develop T cell acute lymphoblastic leukemia by upregulating Notch 1 [8].	
Liver Cancer	TTP/ZFP36	TTP was reduced in HCC cells and tissues. Methylation of a single CpG site within the TGF-beta1-responsive region of the TTP promoter was significantly associated with TTP downregulation in both HCC cells and tissues [53].	
		TTP is downregulated in HCC tumors and hepatic TTP has a tumor suppressive role during tumor progression [54].	
Lung Adenocarcinoma	TTP/ZFP36	Patients with low-TTP expressing lung adenocarcinoma had decreased survival rates and more aggressive tumors [19].	
		TTP is significantly downregulated in human lung tumor samples [37].	
Lymphoma	TTP/ZFP36	MYC suppresses TTP expression and TTP suppression is a hallmark of cancers with MYC involvement. Restoring TTP impairs Myc-induced lymphomagenesis [9].	
	ZFP36L1	ZFP36L1 mediates pro-apoptotic effects in malignant B-cells by promoting the decay of BCL-2 [10].	BCL-2
		Hepatitis B virus associated diffuse large B-cell lymphoma patients have enrichment of genes regulated by ZFP36L1, among others [55].	
Mast Cell Tumor	TTP/ZFP36	Ectopic TTP expression delayed v-H-ras induced mast cell tumor progression by enhancing the degradation of IL-3 [56].	IL-3
Melanoma	TTP/ZFP36	Human melanoma cell lines express very low TTP. TTP regulates the expression of CXCL8 in melanoma cells [57].	CXCL8
		Anti-tumor activity of DM-1, a curcumin analogue in melanoma cells is potentially mediated by TTP, ZFP36L1, and ZFP36L2 among others [58].	
	ZFP36L2	ZFP36L2 was identified as metastasis suppressor, NME1 regulated gene in human melanoma cell lines [42].	
Myelofibrosis	ZFP36L1	ZFP36L1 is a novel candidate tumor suppressor gene in myelofibrosis. Aberrant enhancer hypermethylation of ZFP36L1 reduced its expression in a myelofibrosis cohort [59].	
Pancreatic Cancer	TTP/ZFP36	Pancreatic cancers express a TTP-low gene signature [19].	
		miR-29a was up-regulated and TTP downregulated in pancreatic cancer tissues and cell lines. miR-29a overexpression correlated with increased metastasis. miR-29a enhanced the expression of pro-inflammatory and EMT markers by suppressing TTP [60].	
		TTP was markedly reduced in pancreatic cancer samples. Low TTP was associated with age, tumor size, tumor differentiation, post-operative T, N, and TNM stage. Low TTP predicted poor prognosis in pancreatic cancer patients. Over-expression of TTP in pancreatic cancer cells increased apoptosis, decreased cellular proliferation, and reduced expression of PIM-1 and IL-6 [61].	PIM-1, IL-6
	ZFP36L2	ZFP36L2 was overexpressed and predicted poor patient outcomes in pancreatic ductal adenocarcinoma. ZFP36L2 was regulated by miR-375 in this cancer [62].	
Prostate Cancer	TTP/ZFP36	NEDD9 and ZFP36 were discovered as NF-κB regulators in prostate cancer. NEDD9 and TTP physically interact; knockdown of NEDD9 inhibited prostate cancer cellular proliferation [63].	
		Low-TTP in prostate cancer correlated with increased recurrence. Induced TTP expression reduced cell proliferation, clonogenic growth, and tumorigenic potential of prostate cancer cells [64].	
		TTP protein was significantly lower in human prostate cancer tissues [65].	
		Low TTP levels in prostate cancer shorten time to recurrence or metastasis compared with TTP-high tumors [66].	
Rectal Cancer	TTP/ZFP36	TTP levels in the peripheral blood mononuclear cells were higher in patients with locally advanced rectal cancer that responded to chemoradiation [67].	
Squamous Cell Carcinoma of the Head and Neck (SCCHN)	TTP/ZFP36	Downregulated TTP significantly increased invasion across the basement membrane via IL-6, MMP2, and MMP9 secretion in SCCHN oral-cancer-equivalent three-dimensional in vitro model and the chick chorioallantoic membrane in vivo assay [68].	IL-6, MMP2, MMP9
		Galanin receptor 2 induces angiogenesis in SCCHN through GALR2 ≥ RAP1B ≥ p38MAPK ≥ TTP phosphorylation/inactivation ≥ increased stability of IL-6 and VEGF mRNA, axis [69].	IL-6, VEGF
		TTP increases cisplatin sensitivity of SCCHN cells by inhibiting anti-apoptotic protein BCL-2 [70].	BCL-2
	ZFP36L1	Cisplatin sensitive head and neck squamous cell carcinoma cells have elevated levels of ZFP36L1. Elevated ZFP36L1 blocks cIAP2 and enhances caspase 3 activity [71].	cIAP2
Thyroid Cancer	ZFP36L2	ZFP36L2 was identified as a part of gene-regulatory network involved in endodermal carcinogenesis and validated in cellular and mouse models of thyroid cancer [72].	

## Abbreviations

3′UTR	3′-untranslated region
AHRR	aryl hydrocarbon receptor-repressor
ARES	au-rich elements
BCL2	B-cell lymphoma-2
BERG36	36-Kda Zinc finger protein
BRF	Butyrate response factor
C-MYC	Myc protooncogene
CDK6	cyclin-dependent kinase 6
CIAP2	cellular inhibitors of apoptosis 2
COX2	cyclooxygenase 2
CXCL8	chemokine (c-x-c motif) ligand 8
E2F1	E2F transcription factor 1
EMT	epithelial-mesenchymal transition
ERA	estrogen receptor alpha
ERF	EGF-response factor 1
GALR2	galanin receptor type 2
GOS24	G0/G1 switch regulatory protein 24
HBV	hepatitis b virus
HIF-1A	hypoxia-inducible factor 1 alpha
hTERT	human telomerase reverse transcription gene
IL-13	interleukin 13
IL-23	interleukin 23
IL-27	interleukin 27
IL-3	interleukin 3
IL-33	interleukin 33
IL-6	interleukin 6
IL-8	interleukin 8
LATS2	large tumor suppressor kinase 2
MACC1	metastasis-associated in colon cancer 1
MMP1	matrix metalloproteinase 1
MMP2	matrix metalloproteinase 2
MMP9	matrix metalloproteinase 9
mTOR	mammalian target of rapamycin
NEDD9	neural precursor cell expressed developmentally downregulated protein 9
NF-KB	nuclear factor kappa-light-chain-enhancer of activated B cells
NME1	nucleoside diphosphate kinase 1
NUP475	growth factor-inducible nuclear protein nup475
PD-L1	programmed death-ligand 1
PI3K	phosphatidylinositol-3-kinase
PIM1	proto-oncogene serine/threonine-protein kinase 1
PIM2	proto-oncogene serine/threonine-protein kinase 2
RB	retinoblastoma 1
SASP	senescence associated secretory protein
SNAI1	zinc finger protein snail 1
SOX9	sry-box transcription factor 9
TGF-B1	transforming growth factor beta 1
TIS11	tpa-inducible sequence 11
TNFA	tumor necrosis factor alpha
TTP	tristetraprolin
TWIST1	twist-related protein 1
UPA	urokinase-type plasminogen activator
UPAR	urokinase plasminogen activator receptor
VEGF	vascular endothelial growth factor
XIAP	x-linked inhibitor of apoptosis
ZEB1	zinc finger e-box binding homeobox 1
ZEB2	zinc finger e-box binding homeobox 2
ZFP36	zinc finger protein 36
ZFP36L1	zinc finger protein 36 like 1
ZFP36L2	zinc finger protein 36 like 2
ZFP36L3	zinc finger protein 36 like 3
